# Arthroscopically assisted reduction and internal fixation (ARIF) versus open reduction and internal fixation (ORIF) for lateral tibial plateau fractures: a comparative retrospective study

**DOI:** 10.1186/s13018-019-1186-x

**Published:** 2019-05-24

**Authors:** Marco Verona, Giuseppe Marongiu, Gaia Cardoni, Nicola Piras, Luca Frigau, Antonio Capone

**Affiliations:** 1 Orthopaedic Clinic, Department of Surgical Sciences, Cagliari State University, Lungomare Poetto 12, 09126 Cagliari, Italy; 2 Department Economics and Business Science, University of Cagliari, Cagliari, Italy

**Keywords:** Tibial plateau fractures, Arthroscopic reduction and internal fixation (ARIF), Open reduction and internal fixation (ORIF), Arthroscopically assisted, Schatzker classification, Post-traumatic knee osteoarthritis

## Abstract

**Background:**

This study aims to explore if the arthroscopically assisted reduction and internal fixation (ARIF) technique is superior to the traditional open reduction and internal fixation (ORIF) technique in the treatment of tibial lateral plateau fractures.

**Methods:**

Forty patients with tibial plateau fractures (Schatzker type I–III) treated with ARIF or ORIF from 2012 to 2017 were included in this retrospective study. All patients received pre-operative radiographs and CT scans. The patients were divided into two groups (ARIF or ORIF). All patients had a minimum follow-up of 12 months and an average follow-up of 44.4 months. The clinical and radiographic outcomes were evaluated according to the Knee Society Score (KSS) and the modified Rasmussen radiological score.

**Results:**

Satisfactory clinical and radiological results were found in 39 out of 40 (97.5%) patients. KSS and modified Rasmussen radiological score were significantly better in ARIF group. The mean KSS was 92.37 (± 6.3) for the ARIF group and 86.29 (± 11.54) for the ORIF group (*p* < 0.05). The mean modified Rasmussen radiographic score was 8.42 (± 2.24) for the ARIF group and 7.33 (± 1.83) for the ORIF group (*p* = 0.104). Worst clinical and radiological results were related to concomitant intra-articular lesions (*p* < 0.05). Meniscal tears were found and treated in 17 out of 40 (42.5%) patients. The overall complication rate was 10%.

**Conclusions:**

Both ARIF and ORIF provided a satisfactory outcome for the treatment of Schatzker I–III tibial plateau fractures. However, ARIF led to better clinical results than ORIF. No statistically significant differences were found in perioperative complications, radiological results, and post-traumatic knee osteoarthritis.

**Level of evidence:**

Level III

## Introduction

Tibial plateau fractures are articular lesions that typically involve either active young patients after high-energy trauma or older osteoporotic patients [1–3]. Due to the complexity of injury mechanism, mostly a combination of rotational and axial compression forces, these fractures are often associated with intra-articular lesions such as chondral damage, meniscal tear, and ligament rupture [4–6]. The severity of the fracture pattern is typically characterized according to the Schatzker classification system [7]. Schatzker type I–III fractures involve the lateral tibial plateau and traditionally were treated with open reduction and internal fixation (ORIF) through an anterolateral approach [8]. However, it requires extensive soft tissue dissection and increased risk of post-operative complications has been reported (e.g., infections, hematomas, surgical wound dehiscence, and wound necrosis) [9, 10] even when minimally invasive techniques were proposed for low-grade lateral tibial plateau fractures [11].

Arthroscopically assisted reduction and internal fixation (ARIF), first described by Caspari et al. [12] and Jennings [13], rapidly spread in the last decades as an alternative treatment for low-grade lateral tibial plateau fractures. The main advantage of this technique is that it allows the direct and better vision of articular surface reduction through a less invasive procedure and it also simplifies the treatment of associated intra-articular lesions.

Literature shows good clinical and radiological results at shorts a medium-term follow-up [14–18], particularly for the treatment of Schatzker I–III fractures [19]. Although different authors have suggested that ARIF potentially increases the risk of post-operative compartment syndrome [16, 18], a recent meta-analysis shows lower overall morbidity, better functional outcome, and fewer perioperative complication associated with this technique [20].

Elabjer et al. in 2017, in a RCT of 75 patients with Schatzker I–III fractures, reported excellent clinical and radiological scores in both groups. However, they did not found any statistically significant difference between ARIF and ORIF [21]. Moreover, it is not clear whether the use of ARIF over ORIF reduces the incidence of secondary post-traumatic arthritis in Schatzker type I to III tibial plateau fractures [22].

This study aimed to compare the functional and radiological results and complication rates of arthroscopically assisted reduction and internal fixation (ARIF) with traditional open reduction and internal fixation (ORIF) in the treatment of lateral tibial plateau fractures.

## Material and methods

We retrospectively reviewed a total of 59 consecutive patients with lateral tibial plateau fractures (Schatzker type I–III) surgically treated either by ARIF or ORIF in our Department between January 2012 and December 2017. Exclusion criteria were polytrauma, open fractures, and those cases that required the conversion to ORIF. Patients with significant pre-existing degenerative joint disease, with severe systemic and neurological diseases and patients who did not reach the minimum of 12 months’ follow-up were also excluded. The final study comprised a total of 40 patients divided into 2 groups: 19 in the ARIF group and 21 in the ORIF group. The study was not randomized although treatment and control groups were matched appropriately to reduce selection bias. Institutional review board approval was obtained, and all the patients provided informed consent to participate in the study. This study was performed following the ethical standards of the Declaration of Helsinki. All the patients underwent anteroposterior (AP) and latero-lateral (LL) radiographs of the knee and computed tomography (CT). Fractures were classified according to the Schatzker criteria [7]. Demographic data (gender, age), general risk factors (e.g., hypertension, smoking, diabetes), injury mechanism, and additional intra-articular injuries were collected (Table 1). Mean follow-up was 41.95 months (28.85 SD; range 12–52 months).

**Table 1:** Demographic data of the patients

	ARIF (*n* = 19)	ORIF (*n* = 21)	Total (*n* = 40)	*p* value
Mean age	45.5 (± 17.12)	50.2 (± 14.26)	48 (± 15.67)	0.348
Gender				
Male	9 (47.4%)	12 (57.1%)	21 (52.5%)	0.763
Side				
Right	11 (57.9%)	10 (47.6%)	21 (52.5%)	0.739
Injury mechanism				
Fall	7 (36.8%)	8 (38.1%)	15 (37.5%)	1
Sport	6 (42.1%)	2 (9.5%)	8 (20%)	0.178
Traffic injury	6 (21.1%)	11 (52.4%)	17 (42.5%)	0.313
Pre-operative osteoarthritis				
None	14 (73.7%)	16 (76.2%)	30 (75%)	0.299
Kellgren–Lawrence grade 1	5 (26.3%)	5 (23.8%)	10 (25%)	0.540
Comorbidities				
Smoking	8	5	13/40	0.370
Cardiovascular disease	6	8	14/40	0.921
Diabetes	1	–	1/40	–
HCV	–	2/21	2/40	–

Results are presented as mean values; percentage and SD in parentheses; *p* value set at *p* < 0.05

### Surgical technique

All surgeries were performed by a single surgeon (MV), and the surgical technique was standardized for the group and fracture type. All the patients were positioned supine in general or spinal anesthesia, with 90° of knee flexion and a tourniquet placed on the proximal thigh. In the ARIF group, standard anterolateral and anteromedial ports were used for knee arthroscopy. Joint distension was performed trough fluid inflow by gravity instead of a pump to reduce the risk of compartment syndrome. The first step was the evacuation of the hematoma, and then, the joint was inspected for capsular, ligamentous, chondral damage and meniscal injuries [23]. Split fractures (Schatzker type I) were usually reduced with wide pointed forceps. Fracture’s depression in Schatzker II and III types needs to be elevated; the compressed fragment is centered with an ACL guide, and a drill-tipped guide pin is placed under the bone surface. An angulated bone tamp is then inserted into the drill tunnel in order to elevate the articular surface, and the bony defect is filled with synthetic bone graft substitutes.

**Fig. 1:**
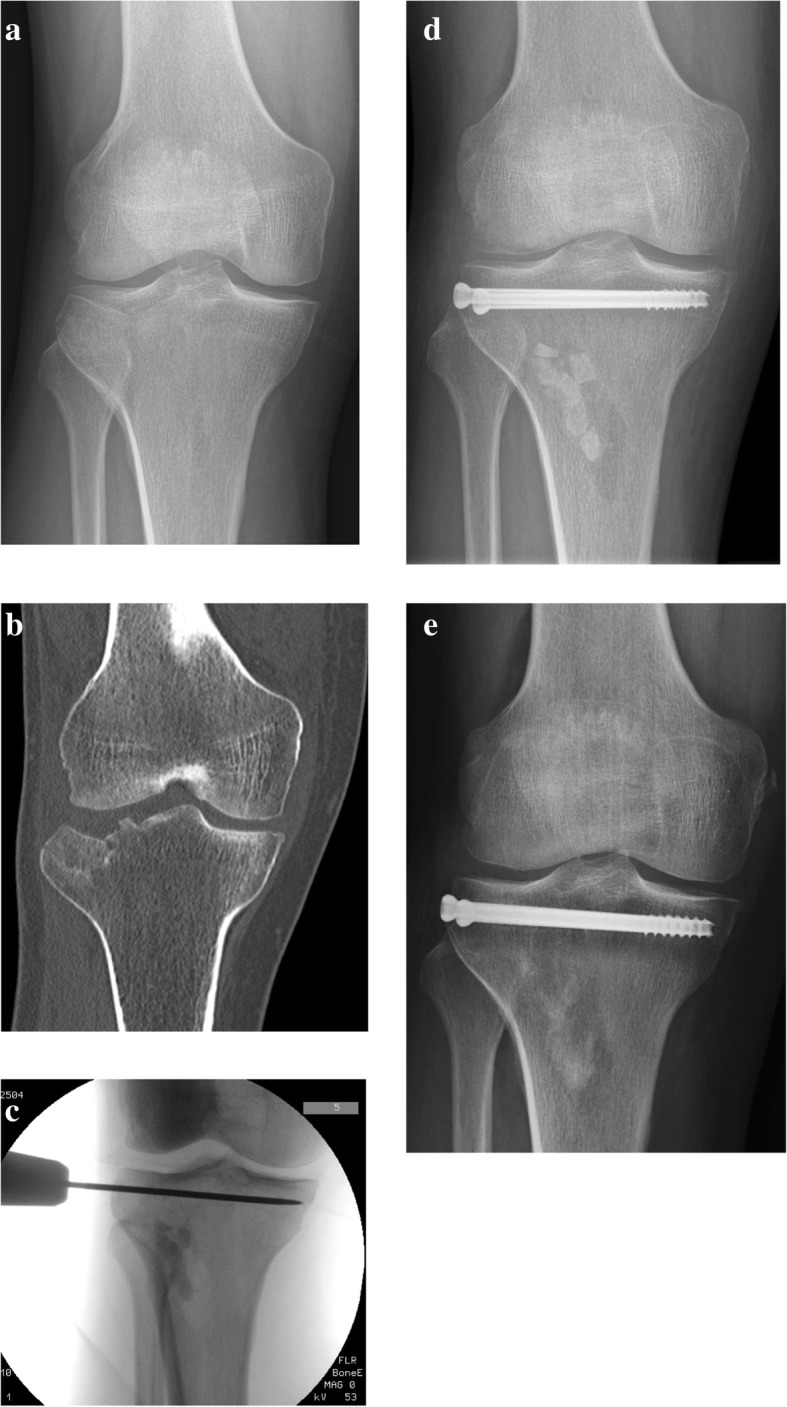
**a** Pre-operative anteroposterior x-rays of a patient with a split compression tibial plateau fracture (Schatzker type III). **b** Coronal section of computerized tomography scan. **c** Intra-operative fluoroscopy showing temporary fixation with a K-wire and metaphyseal bone defect filled with bone substitute grafting. **d** Post-operative anteroposterior x-rays of two cannulated screws fixation. **e** Postoperative 12 months’ anteroposterior x-rays showing good radiological results and initial resorption of the bone graft

In the ORIF group, an anterolateral sub-meniscal approach to the knee was used for joint exposure. Fracture reduction was achieved under direct visualization by opening the lateral fragment to elevate the depressed portion of the articular surface with a bone tamp, and auto- or allograft augmentation was performed. In both groups, internal fixation was performed under C-arm assistance with two or three 6.5-mm cannulated screws or a conventional buttress/locking plate (Fig. 1). Procedures on the additional lesions (e.g., meniscal suture, partial resection, or anterior tibial spine reinsertion) were performed right after the fixation step. The ligament reconstruction procedures for ACL ruptures were postponed after fracture healing.

### Rehabilitation

All the patients received a standardized post-operative rehabilitation protocol. Passive knee motion started on the first post-operative day at up to 90° of flexion. Active knee motion was allowed 7 days after the surgery in both groups and was progressively improved within the initial 8 weeks. Partial weight bearing with the aid of two crutches was allowed after 4 weeks and full weight bearing after 8 weeks, according to radiographic healing of the fracture and pain relief.

### Clinical and radiological outcome evaluation

Anteroposterior (AP) and lateral radiographs were taken on the first post-operative day, at 1 month, 3 months, 6 months, and then yearly. At the final follow-up, standing AP radiographs were taken to assess the overall limb alignment. The evidence of pre-operative osteoarthritis and the possible progression of osteoarthritis grade after the surgery were evaluated according to Kellgren–Lawrence criteria [24].

The patients were evaluated clinically and radiologically using the modified Rasmussen score and the KSS (Knee Society Score) systems [25, 26]. Clinical knee assessment included post-operative ROM evaluation, knee stability, and meniscal tests [27]. Hospital stay and early and late complications were also recorded.

### Statistical analysis

Continuous variables were recorded as mean ± SD. Student’s *t* and chi-square test were used for statistical analysis. For all analyses, a confidence interval level of 95% was selected and statistical significance has been set at *p* values of < 0.05. Linear models were adopted to understand the way that age, sex, KSS, Rasmussen score, Schatzker types, and associated intra-articular lesions were affected by the other features. A feature selection strategy was adopted. All the analyses were performed using the software R 3.4.4.

## Results

A total of 40 patients with Schatzker type I, II, and III tibial plateau fractures treated with ARIF or ORIF were evaluated. There were 21 males (52.5%) and 19 females (47.5%). Mean age at the time of injury was 48 years (± 15.67; range 20–74 years). There were 19 (25.3%) Schatzker type I fractures, 33 (44.1%) type II, and 23 (30.6%) type III, without significant difference in the distribution of Schatzker types among the two groups. In the ARIF group, fixation was obtained with cannulated screws in all the patients. In the ORIF group, 18 patients were treated with plate and screws, the other 3 patients with cannulate screws **(**Fig. 1**)**. Bone defects were filled with bone substitute grafting in all cases. Associated intra-articular lesions were found in 21 out of 40 patients (52.5%), of whom 10 were in the ARIF group and 11 in the ORIF group (*p* = 0.763), see Table 2. Meniscal tears were treated with reinsertion/suture repair in 8 cases (2 ARIF, 6 ORIF; *p* = 0.345); in other 9 cases, partial resection was performed (6 ARIF, 3 ORIF; *p* = 0.337). All the 3 cases of anterior tibial spine avulsion were treated with arthroscopic suture pull-out fixation.

**Table 2:** Fracture classifications, associated intra-articular lesions, and surgical treatment

	ARIF (*n* = 19)	ORIF (*n* = 21)	Total (*n* = 40)	*p* value
Fracture classifications				
Schatzker I	4 (21.1%)	1 (4.76%)	5 (12.5%)	0.281
Schatzker II	8 (42.1%)	8 (38.1%)	16 (40%)	1
Schatzker III	7 (36.8%)	12 (57.14%)	19 (47.5%)	0.334
Treatment				
Cannulated screws	19 (100%)	3 (14.3%)	22 (55%)	< 0.05
Plate	–	18 (85.7%)	18 (45%)	< 0.05
Associated intra-articular lesions				
Meniscal tear	8 (80%)	9 (81.8%)	17 (81%)	0.154
Anterior tibial spine	2 (20%)	1 (9.1%)	3 (14.2%)	0.424
ACL rupture	–	1 (9.1%)	1 (4.8%)	–
Total	10 (100%)	11 (100%)	21 (100%)	0.763
Treatment				
Suture of meniscal tear	2	6	8	0.345
Partial meniscectomy	6	3	9	0.337
Anterior tibial spine fixation	2	1	3	0.925

Results are presented as mean values; percentage and SD in parentheses; *p* value set at *p* < 0.05

The difference in the mean duration of hospital stay was statistically significant: 3.95 ± 1.35 days for the ARIF group and 5.86 ± 4.19 days for the ORIF group (*p* < 0.05).

The overall complication rate was 10%. There were no early or late complications directly associated with arthroscopic procedures in the ARIF group. There was one late deep infection observed in the ORIF group and treated successfully with drainage and i.v. antibiotic therapy. There were three cases of intolerance to the lateral plates, which were then removed after at least 12 months from the surgery. There was not a statistically significant difference in the complication rate between the two groups of patients (*p* = 0.370).

Good clinical and radiological results were obtained in both groups (Tables 3 and 4). There was a statistically significant difference in mean KSS between the two groups (ARIF 92.37 points, ± 6.32; ORIF 86.29 points, ± 11.54; *p* < 0.05). A correlation was found between lower KSS and associated intra-articular lesions (*p* < 0.05).

**Table 3:** Results of clinical evaluation

	ARIF (*n* = 19)	ORIF (*n* = 21)	Total (*n* = 40)	*p* value
KSS	92.37 (± 6.3)	86.29 (± 11.54)	89.17 (± 9.8)	< 0.05
Excellent	19 (100%)	16 (90.5%)	35 (87.5%)	< 0.05
Good	–	3 (14.3%)	3 (7.5%)	0.548
Fair	–	1 (4.8%)	1 (2.5%)	0.625
Poor	–	1 (4.8%)	1 (2.5%)	0.96
SR (%)	19 (100%)	20 (95.2%)	39 (97.5%)	0.928
ROM operated knee	127.89 (± 6.3)	124.76 (± 9.55)	126.25 (± 8.2)	0.348
ROM contralateral knee	132.37 (± 4.2)	133.1 (± 5.12)	132.75 (± 4.7)	0.625
ROM mean difference	− 4.47 (± 5)	− 8.33 (± 9.26)	− 6.5 (± 7.7)	0.106

Results are presented as mean values; SD and percentage in parentheses; *p* value set at *p* < 0.05

**Table 4:** Results of modified Rasmunssen radiological assessment

	ARIF (*n* = 19)	ORIF (*n* = 21)	Total (*n* = 40)	*p* value
Rasmunssen mean score	8.42 (± 2.24)	7.33 (± 1.83)	7.85 (± 2.08)	0.104
Excellent	12 (63.2%)	7 (33.3%)	19 (47.5%)	0.344
Good	4 (21.1%)	8 (38.1%)	12 (30%)	0.448
Fair	–	5 (23.8%)	5 (12.5%)	0.625
Poor	–	1 (4.8%)	1 (2.5%)	0.96
SR (%)	19 (100%)	20 (95.2%)	39 (97.5%)	0.928

*SR* satisfactory results; SD and percentage in parentheses; *p* value set at *p* < 0.05

The differences in term of range of motion (knee flexion) were not statistically significant (Table 3).

The radiological evaluation, according to the Rasmussen score, showed good overall results for both the ARIF group and the ORIF group (mean 8.42 points, ± 2.24 vs. 7.33 points, ± 1.83); the differences between the two groups were not statistically significant (*p* = 0.104). Satisfactory results were reported in 39 out of 40 patients (97.5%); there was only 1 patient of the ORIF group with poor results (< 5 points). Lower Rasmussen radiological scores were related to age, Schatzker III types, and additional intra-articular lesions.

According to the Kellgren–Lawrence criteria, pre-operative osteoarthritis was absent in 30/40 (75%) of the patients, while 10/40 (25%) of the patients were classified as grade I (Table 1). The progression by 1 grade of post-operative osteoarthritis was identified in 7 patients in the ARIF group (36.8%) and 8 in the ORIF group (38.1%). No patients had a postoperative progression of more than 1 grade.

All the fractures were considered radiographically healed within 3 months after the surgery. The patients in the ARIF group underwent to mean 48 days (± 34.04) of post-operative physical therapy without significant differences among the two groups. Full weight bearing was achieved, according to fracture healing and pain relief, after mean 96.6 days (± 63.4). There was a statistically significant difference between the two groups (ARIF 75.5 days, ± 33.87; ORIF 114.8 days, ± 71.11; *p* < 0.05).

## Discussion

The primary goals of the surgical management of tibial plateau fractures are the anatomical reduction and fixation of the articular fracture and the proper treatment of associated intra-articular lesions to achieve early mobilization and reduce the risk of stiffness, instability, and post-traumatic osteoarthritis of the knee [5]. These objectives were traditionally pursued through open reduction and internal fixation with plate and screws, but the last decades’ literature has shown the effectiveness of the arthroscopically assisted treatment [12–18]. Our study aimed to compare the clinical and radiological results of ARIF and ORIF techniques used for Schatzker type I–III fractures in two different groups of patients.

The main advantage of ARIF is that allows the direct vision of articular surface reduction through a less invasive procedure than ORIF and simplifies diagnosis and treatment of associated intra-articular lesions, which typically occurs in 30% to 71% of patients with tibial plateau fractures [4, 6]. In our series, associated intra-articular lesions affected 52.5% of the patients (10/19 ARIF, 11/21 ORIF; *p* = 0.763). All the 17 meniscal tears and the 3 avulsions of the anterior tibial spine, were treated concomitantly to the fixation procedures. Ligament reconstruction for one case of ACL rupture was deferred after fracture healed, as proposed by other authors, in order to avoid time-buying procedures and further complications risk [21, 28].

The difference in the mean duration of hospital stay was statistically significant: 3.95 ± 1.35 days for the ARIF group and 5.86 ± 4.19 days for the ORIF group (*p* < 0.05). It has been addressed to more massive post-operative edema and soft tissue swelling due to ORIF procedures [17, 21].

In a recent meta-analysis, including only RCTs which compared ORIF and ARIF cohorts, overall complication rates ranged from 0 to 26%. The authors reported higher rates of perioperative complications in ORIF than in ARIF patients [20, 29]. We observed an overall complication rate of 10%: 4 late complications (3 fixation intolerance and 1 deep infection) in 40 patients in the ORIF group and no complication in the ARIF group. In our series, the difference was not statistically significant (*p* = 0.370), and it seemed to be related more to the fixation hardware than to the surgical technique itself. Moreover, we did not found any case of compartment syndrome after ARIF procedures. However, the risk of compartment syndrome should be aware the surgeon to use ARIF technique carefully, especially in the medial plateau or bicondylar fractures (Schatzker IV–VI) when a more massive amount of irrigation’s fluid and longer operative times are required. In our current practice, ARIF is, therefore, an exclusive indication for lateral tibial plateau fractures.

Several studies compared the clinical and radiological outcome of ORIF and ARIF procedures. Wang et al. [30] conducted a retrospective analysis of 57 patients with tibial plateau fractures (Schatzker I–IV). The authors concluded that both techniques lead to satisfactory clinical results, but no significant differences in clinical outcome were found. Recently, Elabjer et al. [21], in a randomized controlled trial, evaluated 75 patients with Schatzker I–III fractures. Clinical and radiological scores were excellent in the majority of patients in both groups but without any statistically significant difference. Nevertheless, Sun et al. [20], in a meta-analysis of RCTs, compared clinical and radiological results of the two techniques in Schatzker type I, II, and III fractures. They found statistically significant better clinical outcome and earlier full weight bearing in ARIF than in ORIF. We similarly observed overall satisfactory clinical results in 97.5% of patients. KSS mean score was significantly better in ARIF group than in the ORIF group (92.37 ± 6.3 vs 86.29 ± 11.5; *p* < 0.05). ARIF patients had better mean post-operative knee flexion than ORIF patients, and the loss of ROM, compared to the contralateral knee, observed in ARIF patients was lower than in ORIF patients (− 4.47 ± 5 vs − 8.33 ± 9.26; *p* < 0.05). Moreover, ARIF patients achieved full weight bearing earlier and needed fewer days of physical therapy (*p* < 0.05).

Rasmussen radiological assessment showed good to excellent outcome for both ARIF and ORIF groups. However, our results did not show significant differences between the two surgical techniques and a correlation with the progression of secondary post-operative osteoarthritis. This result is consistent with other reports and could be related to the small sample size and to the relative short minimum follow-up time of our study [20, 21, 29].

Additionally, as reported by other authors, there was no relation between Schatzker types and clinical and radiological outcome [6, 21]. On the other hand, the concomitant diagnosis and treatment of additional intra-articular lesions were associated with both lower clinical and radiological scores.

This study has several limitations. The first limitation is the retrospective design of the study. The second is represented by the relatively small sample of patients enrolled in the study. Third, the minimum follow-up was 12 months and it could be not long enough to observe the development of post-operative osteoarthritis. However, one of the strengths of the study is its case-control design. Another strength of our study is the enrolment of only Schatzker I–III fractures, which share a similar biomechanical characteristic, and the homogeneous distribution of patients’ characteristics (e.g., age, sex, and additional intra-articular lesions) among the two groups.

## Conclusions

In conclusion, both ARIF and ORIF can provide a good clinical and radiological outcome in the treatment of Schatzker type I, II, and III tibial plateau fractures.

However, ARIF patients showed better results in terms of length of hospital stay, clinical scores, and time to full weight-bearing recovery.

Further studies with a prospective design, a large number of participants, and long-term follow-ups are necessary to confirm the effect of ARIF in tibial plateau fractures.

## Abbreviations

ARIF: Arthroscopically assisted reduction and internal fixation
ORIF: Open reduction and internal fixation
KSS: Knee Society Score
ROM: Range of movement
